# Awareness of Female Genital Schistosomiasis: A cross-sectional survey in rural Madagascar

**DOI:** 10.1093/eurpub/ckac131.349

**Published:** 2022-10-25

**Authors:** P Rausche, R Rakotozandrindrainy, T Rasamoelina, RS Rakotomalala, JM Kutz, E Lorenz, J May, RA Rakotoarivelo, DI Puradiredja, D Fusco

**Affiliations:** 1 Department of Infectious Diseases Epidemiology, Berhard Nocht Institute for Tropical Medicine, Hamburg, Germany; 2 German Center for Infection Reserach, Hamburg-Borstel-Lübeck-Riems, Germany; 3 University Antananarivo, Antananarivo, Madagascar; 4 Centre Infectiolologie Charles Mérieux, Antananarivo, Madagascar; 5 Centre Hospitalier Universitaire Androva, Mahajanga, Madagascar; 6 University Fianarantsoa, Fianarantsoa, Madagascar

## Abstract

**Background:**

Infections with S. haematobium are endemic in tropical regions and emerging in some European countries. Prolonged chronic infection with S. haematobium can cause Female Genital Schistosomiasis (FGS), which can lead to serious gynecological conditions, including infertility. However, awareness of FGS is limited, as are adequate guidelines and public health strategies to manage the disease. The aim of this study is to determine the levels and quality of FGS awareness among women and healthcare workers (HCW) in the Boeny region of Madagascar, where the disease is endemic.

**Methods:**

Data collection involved a cross-sectional survey of adult women (n = 694) and HCWs (n = 93) on topics, such as respondents’ sociodemographic background characteristics, FGS awareness, and risk perceptions. Results were analyzed using descriptive statistics including proportions and 95% confidence intervals.

**Results:**

Of the 694 women included in the study, 11.2% (CI [9.0-13.8%]) had heard of FGS. Among these, 34.6% (CI [24.1-46.2%]) were unaware of the gynecological symptoms signature for FGS, and 41% (CI [30.0-52.7%]) were unaware of the urological symptoms of the disease. Out of the 93 HCW surveyed, 53.2% (CI [42.6-63.6%]) had heard of FGS. Among these, 42.0% (CI [28.1-56.8%]) were unaware of the gynecological symptoms, and 52.0% (CI [37.4-66.3%]) were unaware of the urological symptoms.

**Conclusions:**

Preliminary results show overall low levels of FGS awareness among the study population. While more women reported to have never heard of FGS than HCW, the proportion of HCWs who did not know the gynecological and urological symptoms of FGS was higher. Given the occurrence of FGS in Europe and the chronic character of the disease it would be crucial to conduct similar investigations in Europe. Such findings can contribute to the design of targeted local and global FGS awareness campaigns to improve the health of women affected by this disease worldwide.

**Key messages:**

• Awareness of FGS among the study population in Madagascar, an endemic country, is low.

• Lack of awareness can delay the identification of the disease and increase individual and community burden.

